# MicroRNAs in Smoking-Related Carcinogenesis: Biomarkers, Functions, and Therapy

**DOI:** 10.3390/jcm7050098

**Published:** 2018-05-01

**Authors:** Tomomi Fujii, Keiji Shimada, Tokiko Nakai, Chiho Ohbayashi

**Affiliations:** 1 Department of Diagnostic Pathology, Nara Medical University School of Medicine, 840 Shijo-cho, Kashihara, Nara 634-8521, Japan; tokiko@naramed-u.ac.jp (T.N.); ohbayashi@naramed-u.ac.jp (C.O.); 2 Department of Diagnostic Pathology, Nara City Hospital, Nara 634-8521, Japan; k-shimada@nara-jadecom.jp

**Keywords:** microRNA, smoking, carcinogenesis, lung carcinoma, bladder carcinoma

## Abstract

Long-term heavy cigarette smoking is a well-known high-risk factor for carcinogenesis in various organs such as the head and neck, lungs, and urinary bladder. Furthermore, cigarette smoking can systemically accelerate aging, and as the result, promoting carcinogenesis via changing the host microenvironment. Various inflammatory factors, hormones, and chemical mediators induced by smoking mediate carcinoma-related molecules and induce carcinogenesis. MicroRNAs (miRNAs) are a family of short noncoding RNA molecules that bind to mRNAs and inhibit their expression. Cigarette smoke induces the expression of various miRNAs, many of which are known to function in the post-transcriptional silencing of anticancer molecules, thereby leading to smoking-induced carcinogenesis. Analysis of expression profiles of smoking-induced miRNAs can help identify biomarkers for the diagnosis and prognosis of smoking-related cancers and prediction of therapeutic responses, as well as revealing promising therapeutic targets. Here, we introduce the most recent and useful findings of miRNA analyses focused on lung cancer and urinary bladder cancer, which are strongly associated with cigarette smoking, and discuss the utility of miRNAs as clinical biomarkers.

## 1. Introduction

Cigarette smoking has been proposed as the cause of various cancers, including lung, urinary system and bladder, head and neck, liver, esophagus, pancreas, and oral cancer [1,2]. Smoking substantially increases the risk of carcinogenesis and development of cancer in various types of organs [2]. The number of cigarettes smoked per day is positively correlated with clinical outcomes, such as the onset of various carcinomas [1].

The main adverse effect of smoking is that carcinogens derived from cigarettes trigger inflammatory reactions, which lead to damage of tumor-related genes, misrepair, and neoplastic transformations, including autonomy. Aging is also a factor. Immune function is gradually impaired with age, while production of inflammatory cytokines increases. The consequence can be the induction of genetic abnormalities. Smoking-related carcinogenesis is closely associated with aging due to chronic persistent inflammation [3]. The myriad of carcinogens in cigarette smoke includes tobacco nitrosoamine, which may affect tumorigenesis in diverse organs. 4-(Methylnitrosamino)-1-(3-pyridyl)-1-butanone (NNK) is a tobacco-specific metabolite that is a potent carcinogen in experimental animal models of lung adenoma and carcinoma [4,5,6,7,8]. 

MicroRNAs (miRNAs) are short, single-stranded, noncoding RNAs that participate in the post-transcriptional regulation of gene expression [9]. Their primary function is to reduce the expression of their target genes by binding to the 3′ untranslated region (3′-UTR) of the messenger RNA (mRNA), which leads to the degradation of the mRNA or other translational repressive mechanisms. miRNAs are among the most abundant classes of gene regulatory molecules, and thousands of distinct miRNAs have been identified in mammals including humans [10]. The majority of mammalian genes may be under miRNA regulation. At least 45,000 miRNA target sites within human 3′-UTRs are conserved above background levels, and more than 60% of human protein-coding genes are under selective pressure to maintain pairing to miRNAs [11]. miRNAs contribute to biological processes, including the development, maturation, metabolism, aging, and carcinogenesis of various organs. A central goal for understanding the function of all these small regulatory RNAs has been to determine which messages are targeted for repression. 

In addition to lung carcinoma, urothelial carcinoma in the urinary tract is among the most common malignancies in individuals with a history of heavy smoking. However, even though smoking is considered to be a common risk factor of urothelial and lung carcinomas, it remains unclear how smoking and its related molecular signals can initiate or promote carcinogenic processes in the urinary tract as compared with lung carcinoma. To date, reviews have focused only on comparisons between urothelial and lung carcinoma relative to cigarette smoking. Previous studies revealed that cigarette smoking regulates key miRNAs involved in the expression and regulation of target genes in lung carcinoma [12,13,14,15,16]. In addition, molecular markers associated with cigarette smoking have been found in urothelial carcinomas [17,18,19]. These reports have raised the possibility that cigarette smoking may contribute to oncogenic or antioncogenic gene expression by regulating miRNAs in urinary tract cancer. 

In this review, we summarize the evidence concerning the smoking-dependent regulation of miRNA and its target genes in lung and urinary tract carcinomas. We also propose available methods or evidence to elucidate the mechanisms of tumorigenesis and progression of urothelial carcinomas.

## 2. Correlation among Smoking, Cancer, and Expression of miRNAs

Environmental factors, including diet, smoking, alcohol consumption, stress, infectious agents, and environmental carcinogens, are important in the pathogenesis of cancers through epigenetic modifications [20]. Increasing epidemiological evidence links cigarette smoking with cancer. Cigarette smoking and infectious agents are the major causes of cancer in Japan [21]. Evidence of the association between cigarette smoking and cancers of the respiratory system (including lung), digestive tract, and urinary system has accumulated over time [1]. A comprehensive meta-analysis quantified much of the existing evidence linking smoking with well-known anatomic cancer sites, such as the respiratory, upper digestive, and urinary tracts [1].

Carcinogenesis is a multistep process that includes the initiation, promotion, progression, malignant conversion, and finally the formation of a fully developed tumor in which genetic changes, including some kinds of molecular function failure based on DNA breaks, play critical roles [22]. At variable steps of DNA breaks, the aberrant expression patterns of miRNAs can be oncogenic or antioncogenic factors [23] and are associated with cellular dysfunction and onset of disease [12]. Altered miRNA expression causes neoplastic transformation [24]. In non-small cell lung carcinoma (NSCLC), miR-224 promotes cell migration, invasion, and proliferation. miR-224 directly targets tumor necrosis factor-alpha-induced protein 1 and SMAD4, and the expression of miR-224 is regulated by extracellular signal-regulated kinase signaling through the binding of c-Jun binding to the miR-224 promoter region in lung carcinoma [25]. Immunoglobulin binding protein 1 (IGBP1) is commonly expressed in lung adenocarcinoma, including the early stage of lung adenocarcinogenesis. miR-3941 is a tumor-suppressive miRNA that directly targets and regulates IGBP1. Overexpression of miR-3941 and suppression of IGBP1 induces apoptosis by events that include increased cleavage of caspase-3 and poly (ADP-ribose) polymerase [26]. Alterations in the expression of miR-125b, a tumor-suppressing miRNA, trigger an early event in prostate carcinoma. miR-125b directly targets ErbB2/B3 and MET, and loss of miR-125b results in enhanced signaling via the MET-regulated phosphoinositol-3-kinase/protein kinase B and RAS/pMEK pathways [27]. 

Aging manifests biologically as changes in genome and protein instability, telomeric shortening, nutrient-sensitive abnormalities, mitochondrial dysfunction, cellular senescence, stem cell depletion, and intercellular signal transduction abnormalities [28,29,30]. These molecular dysfunctions mediate apoptosis, cell senescence, and/or autophagy or carcinogenesis by aberrant genomic expression. Various carcinogens produced by smoking accelerate aging accompanied with genomic and molecular changes through genetic modifications or mutations [31,32,33,34]. Oncogenic miRNAs inhibit the expression of some tumor suppressor molecules and promote the acceleration of aging with associated carcinogenesis; in contrast, suppression of these miRNAs promotes cellular senescence and decreases cell proliferation [2,11,35].

Some miRNAs play an important role in tumor invasion and metastasis [36]. The epithelial-mesenchymal transition (EMT) is a crucial mechanism for tumor invasion and metastasis. We previously demonstrated that the miR-331-3p and syndecan-1 axis may contribute to the progression of prostate cancer through the regulation of nucleus accumbens-associated protein 1 and neuropilin 2, and the promotion of EMT [37]. The overexpression of miR-331-3p in asbestos-related lung cancer has been described [38]. The collective evidence demonstrates that miR-331-3p is a tumor-promoting miRNA because it enhances the EMT. Upon binding of programmed cell death protein 1 (PD-1), a cell surface receptor present on T cells and pro-B cells, to its ligands PD-L1 and PD-L2, an immunosuppressive effect is triggered, which allows tumor cells to escape immune detection and destruction [39]. PD-L1 is highly expressed in multiple intracarcinoma cell types that may play an effective role in CD8+ T-cell suppression. This has profound therapeutic effects in advanced NSCLC, such as the disturbance of interactions between PD-1, which is a coinhibitory receptor, and PD-L1, which is a ligand [40]. miR-200 acts as the target of the EMT-inducing transcription factor zinc finger E-box-binding homeobox 1 (ZEB1) in the regulation of gene expression of PD-L1. miR-200 also controls cytotoxic CD8p tumor-infiltrating lymphocytes, and the miR-200/ZEB1 axis also regulates the expression of PD-L1. Thus, an EMT regulatory program for the exhaustion of CD8p tumor-infiltrating lymphocytes and immunosuppression of PD-L1 via the miR-200/ZEB1 axis is critical to tumor metastasis. Likewise, miR-155 functions as an immune system activator by blocking autophagy, which promotes the upregulation of CD4+ and infiltration of FoxP3+ tumor-infiltrating lymphocytes, which results in increased lung carcinoma chemosensitivity [41].

### 2.1. Lung

Lung cancer is a major neoplasia. There are two main distinct histological types: small cell and non-small cell carcinomas (NSCLC) [42]. Small cell carcinoma is completely different from NSCLC from the perspectives of pathogenesis, cellular origin, molecular alteration, and histopathological and clinical features [43,44]. On the basis of molecular pathology, lung carcinoma occurs after multiple processes including genomic instability, mutations, deletions, insertions, modifications (such as methylation and sialylation), and gene silencing by miRNAs [13,45]. During the early steps in the oncogenesis of lung carcinoma, mutations of tumor suppressor or enhancer genes have been identified. Once the repair of DNA damage fails, these intrinsic mutations may be important carcinogens underlying genomic instability and may accelerate cancer progression [46].

As well as genetic modification, epigenetic coregulation of miRNAs and their target molecules is an important step. miRNA-486-5p is a tumor suppressor miRNA that is often downregulated, and which correlated with ankyrin 1 (ANK1) expression in NSCLC. The ANK1 promoter CpG island is unmethylated in normal lungs, but is methylated in the lungs of individuals with adenocarcinoma, and more so than in those with squamous cell carcinoma [47]. Among exogenous carcinogens, cigarette smoking is the most important risk factor for the onset and development of lung cancer. Both smokers and those exposed to secondhand smoke in households or workspaces are at a higher risk of lung carcinoma than nonsmokers [46,48]. Exposure of more than pack-years increased the risk of lung cancer among nonsmokers by approximately 30% [48,49]. ANK1 methylation is significantly more prevalent in adenocarcinoma compared to squamous cell carcinoma [47]. E-cadherin expression levels in smokers with non-small cell lung cell carcinoma are reportedly lower than those in people with the disease who had never smoked. Cigarette smoking induces the repression of E-cadherin by regulating the expression of transcription factors lymphoid enhancer-binding factor 1 and Slug, which leads to EMT [8]. Cigarette smoking for many decades leads to genetic mutations or deletions of bronchial epithelial cells, which can result in dysplasia and subsequent carcinoma. Smoking affects the expression of miRNAs in the bronchial epithelium. Gene mutations most closely related to carcinogenesis occur due to exposure to some cigarette carcinogens. A spectrum of TP53 mutations have been documented in cancers arising in active as well as former smokers [50,51], with greater prevalence in women with a history of heavy smoking. In addition, the significance of the genetic background for lung carcinoma has been noted, with miRNA-related molecular signals mainly involved in malignant transformation. For example, we introduced miR-126, which plays a crucial role as a cancer-suppressive miRNA in the liver, pancreas, lung, colon, and stomach [52,53,54,55,56,57]. In lung carcinoma cells, including cell lines, miR-126 expression is suppressed, leading to the upregulation of the target gene encoding vascular endothelial growth factor [58]. Significant changes have been found in the expression of 133 miRNAs in the lungs of rats exposed to cigarette smoke; some of these miRNAs may participate in the carcinogenic process [59]. In vitro experimental model systems for miRNA alterations mediated by cigarette smoking in normal human bronchial epithelial and lung cancer cells derived from smokers and nonsmokers have demonstrated the repression of miR-487b, which leads to the upregulation of suppressor of zest 12 (SUZ12), B cell-specific Moloney murine leukemia virus insertion site 1 (BMI1), wingless-type family member 5A (WNT5A), MYC, and KRAS, thereby increasing cell proliferation, invasion, tumorigenicity, and metastatic potential of lung cancer cells in the cigarette-smoking group [14]. In smokers, the expression of antioncogenic let-7 family members is inversely associated with the number of cigarettes smoked per day by females, but not by males [13]. Expression of oncogenic miR-21 correlates with the number of cigarettes smoked per day in squamous cell carcinoma, but not in adenocarcinoma [13]. Interestingly, in one study, no significant differences were observed in smoking histories, such as current versus former smoking status, years of smoking, age at smoking initiation, or exposure to passive smoking [13]. The microarray analysis of RNA in bronchial epithelial cells revealed a significant difference between smokers and nonsmokers in the expression of 28 miRNAs [15]. Interestingly, in females, smokers have a higher risk for epidermal growth factor receptor (EGFR) mutations (exons 18, 19, and 21) compared to nonsmokers. These findings suggest that the histological type is closely associated with the mechanism of action of smoking-related carcinogens. There are two mechanisms: One is smoking-related and -unrelated carcinogenesis and cancer development integrated by miRNA. The other is EGFR mutations based on the DNA modification and mutations in lung cancer. Moreover, detailed information of the smoking history allows us to select biomarkers and the most useful cancer therapy.

### 2.2. Urinary Tract

Various risk factors for urothelial carcinoma have been reported. They include cigarette smoking and occupational exposure to aromatic amines, polycyclic aromatic hydrocarbons, or dyes; alcohol or tea consumption; treatment with medical compounds; and genetic susceptibility [19]. Cigarette smoke contains more than 4000 identified chemical constituents that are associated with a high risk for cancer throughout the urinary tract [19,60]. Cigarette smoke contains polycyclic aromatic hydrocarbons, aromatic amines, and N-nitroso compounds [61,62]. These carcinogens induce the formation of DNA adducts and cause tumorigenesis of the bladder. Therefore, the level of DNA adducts is a useful biomarker to understand the degree of exposure to cigarette smoke-related carcinogens. Case-control studies have reported the higher incidence of renal pelvis and ureter cancers compared to bladder cancer, because exposure to carcinogens may be more concentrated in the renal pelvis and ureter than the bladder, where dilution or degradation of carcinogens may occur [19].

Some mechanisms of carcinogenesis in the urinary tract have been suggested. For example, DNA methylation retains cell proliferation in the normal urothelium [63,64,65,66]. One group of gene clusters was estimated taking into account the important role of miRNAs. miR-99a was found to be overexpressed in tumors with muscle invasion of the bladder. Marked downregulation of miR-99a and miR-205 was observed in smokers with bladder cancer [67]. Additionally, a significant association was detected between smoking and muscle invasion of the bladder [59]. 

**Figure 1:**
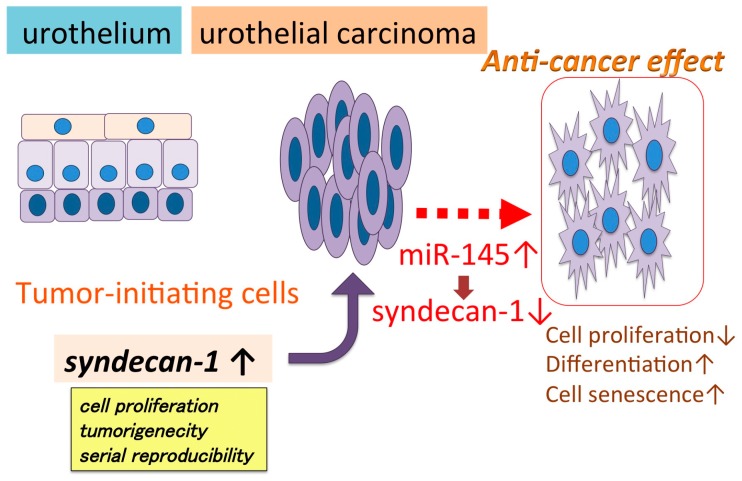
miR-145 regulates cell proliferation, differentiation, and senescence by regulating syndecan-1 in urothelial carcinoma cells. miR-145 and syndecan-1, a putative direct target of miR-145, control the expression of cell differentiation markers [70].

Many reports [68,69,70,71,72,73,74,75,76,77,78,79,80,81,82,83,84,85,86,87,88,89,90,91,92,93,94,95,96,97,98,99,100,101,102,103,104,105] have indicated the key role of miRNAs in neoplastic transformation by propagating cell proliferation and tumor invasion and migration (Table 1, Figure 1). Our previous study indicated that miR-145 suppresses syndecan-1, upregulates stem cell factors, and induces cell senescence and differentiation, and increased squamous, glandular, and neuroendocrine markers. These results suggest that miR-145 utilizes syndecan-1 to modulate cell proliferation, reprogramming, and differentiation in urothelial carcinoma [86] (Figure 1). However, we have limited data on the interaction of miRNA with high-risk compounds, such as cigarette smoke, for carcinogenesis. Cigarette smoking greatly contributes to urothelial carcinoma, and it is reported that cigarette smoking cessation decreases the risk of urothelial carcinoma and may contribute to preventing recurrence and progression. Smoking-induced carcinogenesis may be different from the miRNA-related tumor extension mechanism that has been reported. Thus, miRNA expression associated with smoking-related carcinogenesis may be different from the mechanism of progression-related miRNA expression. If the miRNAs associated with carcinogenesis related to smoking can be determined, it could help in the elucidation of more detailed carcinogenetic mechanisms and detection of early stages of urothelial carcinomas. New findings of miRNAs involved in molecular abnormalities caused by smoking may lead to the development of liquid biopsy techniques using blood and urine for the diagnosis of cigarette-smoking related tumors, including lung and urothelial carcinomas.

## 3. Relationship between Smoking and DNA Breaks

Cigarette smoke is a rich source of polycyclic aromatic hydrocarbons, aromatic amines, heterocyclic amines, and *N*-nitroso compounds, and these compounds may lead to DNA damage. Additionally, cigarette smoke is also a rich source of reactive oxygen species, which can induce damage to DNA and can accumulate in the urinary bladder as metabolic products of the chemical carcinogens of cigarette smoke [31,32]. The constituents of cigarette smoke can cause DNA double-strand breaks, leading to tumorigenesis. Nibrin (NBS1), a component of the MRE-RAD50-NBS1 (MRN) gene, plays an important role in the DNA double-strand break repair pathway [22]. NBS1 has been found to increase NBS1 gene expression and associated NBS1 polymorphisms in smoking-related cancers, such as those of the lungs, liver, esophagus, head, and neck [106,107]. 

These findings support the hypothesis that genetic variation plays a critical role in smoking-related carcinogenesis.

## 4. Smoking and Aging

During aging, activation of various steps of the carcinogenic process occurs. These include mutation, induction, and cell proliferation. Cigarette smoking has cancer-promoting effects that include the induction of mutations. Cigarette smoking induces many steps of carcinogenesis. If cigarette smoking affects all carcinogenic processes in the same proportion to aging, the risk prediction in current and former smokers can be an efficient epidemiological cancer model. The duration of cigarette smoking is a more powerful predictor of lung carcinoma mortality than cigarette smoking intensity, regardless of age and sex [108].

**Figure 2:**
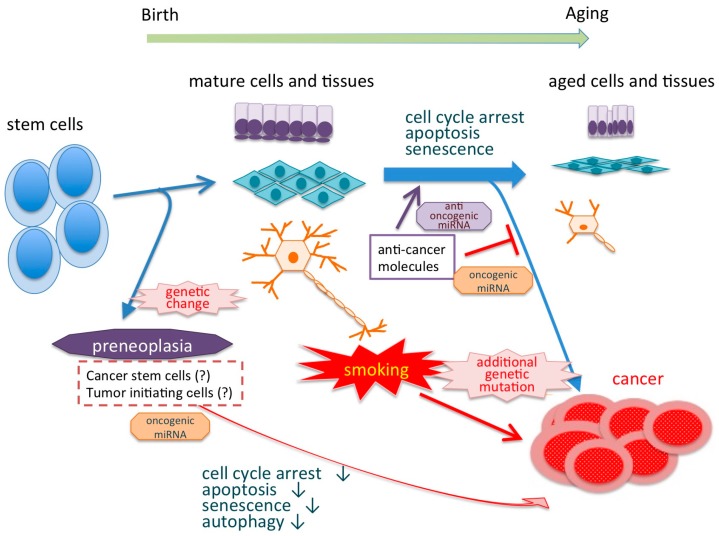
Life time from stem cells to aging and/or carcinogenesis. Genetic changes such as mutations induce carcinogenesis by suppressing the production of anticancer molecules via activating oncogenic miRNAs.

Regardless of smoking, most cancers occur in adults older than 70 years of age. However, many cancers, including lung, gastrointestinal, and urothelial carcinoma, occur in younger adults because of cigarette smoking. Aging has various genomic and epigenomic features, such as increased genomic instability, shortened telomeres, global DNA hypomethylation, complex histone modification, and deregulation of miRNA expression. Features of genetic change, cellular dysfunction, and carcinogenesis in aging cells are biologically similar to the characteristics observed during the carcinogenesis arising from cigarette smoking [28] (Figure 2). For example, autophagy and cell senescence have a dual role in carcinogenesis. They both have tumor growth-promoting and tumor-suppressive effects. Autophagy is downregulated in precancerous lesions and cancers. However, autophagy is initiated to respond to smoking-induced oxidative damage, which can have a protective effect in the early stages of carcinogenesis [109]. In our study, miR-331-3p upregulated autophagy via increasing LC3 concomitantly with a reduction of cell proliferation in urothelial cell lines (unpublished data). Determination of the miRNAs controlling the biological pathways that greatly contribute to aging, such as autophagy and cell senescence, may reveal the mechanism underlying smoking-related carcinogenesis.

## 5. MiRNA Detection 

Blood- and urine-based biomarkers for circulating miRNAs can be detected using several tools, such as quantitative reverse transcription PCR (qRT-PCR) [110], microarray [111], deep sequencing [112], and next-generation sequencing (NGS) [112] in various tumors [113]. miRNAs are stably expressed in human blood, and circulating miRNAs may serve as novel molecular markers for tumors [114,115,116]. An analysis of the expression of miRNAs in plasma from ALK-positive NSCLC patients identified mir-660-5p and miR-362-5p as potential predictors for the response to crizotinib treatment [114]. The detection of circulating miRNAs in plasma and other body cavity fluids is a more useful tool for noninvasive biomarkers than other tissue or cytology specimens for the evaluating of the therapeutic response or to predict outcome [114,115,116].

High-quality nucleic acid extraction is necessary to obtain better sensitivity and specificity in the detection of tumor-specific RNA. The liquid-based cytology and tissue specimens treated with formalin-based fixation are unsuitable for extraction of DNA and/or RNA. We previously reported that the quantity of nucleic acid derived from cancer cells fixed using formaldehyde-containing fixatives is significantly lower compared to alcohol-based fixatives [117]. Since liquid biopsy specimens do not require formalin fixation, high-quality nucleic acid can be extracted. It is important to choose the method based on the detection-related purpose of the extracted nucleic acid extraction to the detection tool.

## 6. Conclusions

Various genes have been associated with the neoplastic transformation of respiratory epithelium by cigarette smoking. miRNAs play an important role in the regulation of genes involved in carcinogenesis and cancer progression, such as those involved in autophagy and cell senescence. In addition, a number of reports have shown that miRNAs contribute to the genetic change caused by cigarette smoking. Thus, miRNAs appear to be a mainstay of smoking-induced cancer development in the lung. Similar mechanisms involving aberrant expression and mutations of genes due to smoking, leading to urothelial carcinoma in the urinary tract, have been reported (Table 1). However, the relationship between the cigarette smoking-related regulation of miRNA and carcinogenesis remains unknown. Chemical compounds produced by smoking cause biological influences via aberrant expression and mutation of miRNAs. Therefore, cancer-related molecules controlled by miRNA are involved in carcinogenesis and cancer progression and could be the leading cause of smoking-related carcinogenesis. miRNA and downstream molecules could be considered as biomarkers, and it is a major future goal to consider these biomarkers in the development of molecular target therapy. Smoking cessation is expected to improve the prognosis or prevent the recurrence or (temporally heterogeneous) multiple occurrences of urinary tract cancers.

The molecular mechanisms of smoking-related carcinogenesis in relation to miRNA have been well established in lung cancer, but many unknown issues and problems need to be addressed in urothelial carcinomas because smoking is a high-risk factor for these types of cancers. We need to examine the association between urothelial carcinoma and smoking. The duration of cigarette smoking is associated with biological genetic features and carcinogenesis-related changes similar to aging. The promotion of the aging effect by cigarette smoking is responsible for the occurrence of carcinogenesis in adolescence or young adults (Figure 2). Estimation of miRNAs and their target molecules associated with smoking provides useful information to develop a novel cancer prevention tool, diagnostic markers, and molecular target therapy.

## Figures and Tables

**Table 1:** Relationship between the microRNA (miRNA) and mRNA of target molecules.

miRNA	Target Molecules	Function	Reference
miR-99a-5p	mTOR	Decreased phosphorylation of mTOR and AKT	[68]
miR-29a	DNMT3A, DNMT3B, MAT2A, SMS	Involvement in the cysteine and methionine metabolism	[69]
miR-210	HIF-1α	Promoting upper tract urothelial carcinoma carcinogenesis	[70,71]
miR-429	CDKN2B	Promoted cell growth and decreased apoptosis	[72]
miR-30a-5p	claudin-5	Suppressed cell proliferation, migration and EMT	[73]
miR-32-5p, -224-5p, -412-3p, -203a-3p, -205-5p		Cancer specific survival, tumor progression, EMT	[74]
miR-21-5p		Novel biomarker of urothelial carcinoma in urine	[75]
miR-193b	ETS1, Cyclin D1	Inhibited cell migration activity, arrested cell at G1 phase; sensitized CDDP treatment	[76]
miR-3713	MMP9	Control of cell invasiveness	[77]
miR-451	c-Myc	Suppressed cell migration and invasion	[78]
miR-497	E2F3	Inhibited cell proliferation, migration and invasion	[79]
miR-877-3p	p16	Increased the expression of p16, inhibited cell proliferation and tumorigenicity	[80]
miR-130b	NF-κB	Persistent activation of NF-κB; promote the malignant progression of urothelial carcinoma	[81]
miR-133b		Novel biomarker of urothelial carcinoma in the tissue	[82]
miR-146a-5p		Novel biomarker of urothelial carcinoma in urine	[83]
miR-429	E-cadherin	Decreased cell migration and invasion through reducing ZEB1 and β-catenin	[84]
miR-30a	Notch1	Decreased cell proliferation and migration, activated cell cycle arrest	[85]
miR-145	syndecan-1	Suppressed cell proliferation, induced cell senescence, differentiation	[86]
miR-24	CARMA3	Inhibited cell proliferation, invasion and EMT	[87]
miR-148a	DNMT1	Reduced cell viability through apoptosis	[88]
miR-182		Novel biomarker of urothelial carcinoma in urine	[89]
miR-9	CBX7	Decreased cell invasion ability	[90]
miR-34a	S100P	Decreased cell invasion ability	[91]
miR-100	BAZ2A, mTOR, SMARCA5	Increased cell proliferation, anti-apoptosis	[92]
miR-99a, -100	FGFR3, FOXA1	Associated with regional hypomethylation	[93]
miR-1	UCA1	Decreased cell proliferation and motility, induced apoptosis	[94]
miR-29c	BCL-2, MCL-1	Induced apoptosis	[95]
miR-101		Novel biomarker of urothelial carcinoma in the tissue	[96]
miR-126	ADAM9	Decreased cell invasion	[97]
miR27a	AGGF1	Regulation of hypoxia-induced apoptosis	[98]
miR-320a	ITGB3.	Decreased cell invasion ability	[99]
miR-23b	Zeb1	Inhibited cell proliferation, induced G0/G1 cell cycle arreset	[100]
miR-96	FOXO1	Tumorigenesis, control cell apoptosis	[101]
miR-34a	Notch1	Decreased cell invasion and migration	[102]
miR-143	cyclooxygenase-2	Decreased cell proliferation and motility	[103]
miR-125b	E2F3	Regulate G1/S transition through the E2F3-cyclin A2 signaling pathway	[104]
miR-101	EZH2	Inhibited cell proliferation and colony formation	[105]
